# The Role of Carrion Supply in the Abundance of Deep-Water Fish off California

**DOI:** 10.1371/journal.pone.0049332

**Published:** 2012-11-02

**Authors:** Jeffrey C. Drazen, David M. Bailey, Henry A. Ruhl, Kenneth L. Smith

**Affiliations:** 1 Department of Oceanography, University of Hawaii, Honolulu, Hawaii, United States of America; 2 University of Glasgow, Institute for Biodiversity, Animal Health and Comparative Medicine, Glasgow, United Kingdom; 3 National Oceanography Centre, University of Southampton, Waterfront Campus, Southampton, United Kingdom; 4 Monterey Bay Aquarium Research Institute, Moss Landing, California, United States of America; Institute of Marine Research, Norway

## Abstract

Few time series of deep-sea systems exist from which the factors affecting abyssal fish populations can be evaluated. Previous analysis showed an increase in grenadier abundance, in the eastern North Pacific, which lagged epibenthic megafaunal abundance, mostly echinoderms, by 9–20 months. Subsequent diet studies suggested that carrion is the grenadier's most important food. Our goal was to evaluate if changes in carrion supply might drive the temporal changes in grenadier abundance. We analyzed a unique 17 year time series of abyssal grenadier abundance and size, collected at Station M (4100 m, 220 km offshore of Pt. Conception, California), and reaffirmed the increase in abundance and also showed an increase in mean size resulting in a ∼6 fold change in grenadier biomass. We compared this data with abundance estimates for surface living nekton (pacific hake and jack mackerel) eaten by the grenadiers as carrion. A significant positive correlation between Pacific hake (but not jack mackerel) and grenadiers was found. Hake seasonally migrate to the waters offshore of California to spawn. They are the most abundant nekton species in the region and the target of the largest commercial fishery off the west coast. The correlation to grenadier abundance was strongest when using hake abundance metrics from the area within 100 nmi of Station M. No significant correlation between grenadier abundance and hake biomass for the entire California current region was found. Given the results and grenadier longevity, migration is likely responsible for the results and the location of hake spawning probably is more important than the size of the spawning stock in understanding the dynamics of abyssal grenadier populations. Our results suggest that some abyssal fishes' population dynamics are controlled by the flux of large particles of carrion. Climate and fishing pressures affecting epipelagic fish stocks could readily modulate deep-sea fish dynamics.

## Introduction

Abyssal fishes live in the largest habitat on earth. Many are predators and scavengers [1], [2], [3], [4] so they may substantially influence community dynamics across trophic levels over large areas of the planet. Top predators have been shown to alter the abundances and behaviors of their prey populations, exert selective pressures, alter biodiversity and alter biogeochemical cycling in both marine and terrestrial systems [5], [6], [7], [8], [9]. Much of our understanding of top down effects in ecosystems is derived from time-series of abundance and/or fishing data. The abundance or distributions of top predators in marine ecosystems also have been shown to be controlled by interannual scales of climate forcing such as El Nino and the PDO [10], [11], [12] and frequently long term reductions are observed as a consequence of fishing. Few time series of deep-sea systems exist from which the factors affecting abyssal fish populations can be evaluated.

A unique abyssal site, Station M, in the eastern North Pacific has been comprehensively studied over more than two decades [13], [14]. The dynamics of life at the seafloor are strongly linked to the flux of particulate organic matter generated in surface waters. Seasonal and interannual increases in fluxes result in increases in sediment community activity and macrofaunal abundance, biomass, and average size and changes in the rank order of the most important taxa [15], [16]. The larger epifaunal megafauna, principally echinoderms, show similar changes at interannual time scales, lagging the particulate flux by 11–22 months [17], [18].

At Station M, abyssal fishes have been shown to increase three fold in abundance over a 15 year period [19]. The dominant fishes are two species of grenadier (Macrouridae), *Coryphaenoides armatus* and *C. yaquinae*, which comprise 97% of the fishes observed in camera sled transects and baited camera deployments. Their abundance was significantly correlated to that of the mobile epibenthic megafauna (mostly echinoderms) but not to surface climate indices used to predict production or to direct estimates of food supply, particulate organic carbon (POC) flux. Many grenadiers are slow growing and can reach ages of ∼75 years [20], [21]. Thus the changes in abundance that were observed almost certainly are driven mainly by migration in response to variation in food availability. Indeed the abundance of epibenthic echinoderms was correlated to bentho-pelagic fish abundance [19]. However, later work by Drazen et al (2008), showed that the two fishes at this site eat very few echinoderms with as much as 69% of the diet of larger *C. armatus* composed of epipelagic carrion [1]. The carrion consisted of the remains of epipelagic fishes, principally jack mackerel and hake, and gonatid squids. The stomach content analysis was corroborated by stable isotope analysis of the grenadiers and their prey sources, again suggesting that carrion was the most important food type [1]. Fatty acid biomarker work examined the potential for the fishes to consume different prey and found that while carrion and benthic crustaceans were consumed that there was no evidence for substantial consumption of echinoderms, which had distinct fatty acid signatures [22].

Our goal was to evaluate if changes in carrion supply might drive the temporal changes in grenadier abundance at Station M. Several studies have indicated shifts in nekton abundance in the CA current system. For instance Zeidberg and Robison [23] showed the dramatic appearance of humboldt squid, *Dosidicus gigas*, off central California and persistence in the system from 2002 onwards. Brodeur et al [24] have shown regional shifts in the nekton community structure between 1998 and 2002 related to a shift in the sign of the Pacific Decadal Oscillation. We have analyzed a unique 17 year time series of abyssal grenadier abundance in conjunction with abundance estimates for surface living nekton eaten by the grenadiers as carrion. Our results show a positive correlation to Pacific hake, the most abundant nekton species in the region and the target of the largest commercial fishery off the west coast [25], [26], [27]. Our results suggest that, unlike other benthic community groups, some abyssal fishes' population dynamics are controlled by the flux of large particles of carrion.

## Methods

### Ethics Statement

The research conducted for this project was done in accordance with approved protocols (07–039) from the University of Hawaii, Institutional Animal Care and Use Committee.

### Approach

To assess the relationship between carrion supply and grenadier abundance we correlated the grenadier time series to three metrics of nekton abundance. Direct estimates of standing stocks of carrion are not possible because the abyssal scavenging fauna rapidly removes this food from the seafloor [28], [29] and visual observations are rare, even for large persistent carrion parcels such as dead whales [30], [31], [32]. We focused our attention on time series of abundance of nekton that were important carrion sources to the grenadiers as determined from diet studies [1]. These are gonatid squids, jack mackerel (*Trachurus symmetricus*) and Pacific hake (*Merluccius productus*). The latter makes up 61% of the pelagic fish biomass in the California Current ecosystem [26].

### Grenadier data

Grenadier abundance was estimated using 54 towed camera transects from 1989 to 2007. The study area is known as Station M (34° 50′ N, 123° 00′ W), and consists of a 25×35 km area of the seafloor on the Monterey fan at 4100 m depth, 220 km offshore of Point Conception, California. [13], [14] [34° 50′ N, 123° 00′ W; 13,14].

The camera system and the methods used to estimate fish density have already been described in detail elsewhere [19], [33]. In short, the camera system was towed along the seafloor at an average speed of 0.8 m s^−1^ for a mean distance of 1254 m taking images every 4–5 s. The area viewed was the product of the distance of the transect and the effective transect width based on fish visibility across the transect axis [19], which varied between deployments due to lighting intensity and film development. Transects conducted within a month of each other (during the same field event) were considered replicates and density estimates were averaged. This yielded 39 time point estimates across the study period.

Size frequency distribution of the grenadiers was also determined. This was not possible using the towed camera because of the variable position and orientation of the fish above the seafloor. Downward looking baited cameras were used 10 times from 1990–1992 [34] and 3 times in 2005. In 2005 the “Sprint” video lander was used, recording 1 min video sequences with 4 min intervals between them. The characteristics of this lander have been described elsewhere [35], with the only modification being the removal of the electrical stimulation system so that fish could feed at the bait undisturbed. Fish total length was measured by taking individual frames from the video sequence and digitizing along the centre line of fish using Image J. From 1995–1998 grenadiers were captured during 8 sampling events using baited traps and longlines [36]. Fish total and pre-anal fin length were measured directly and their wet weight was estimated from existing length weight relationships for *Coryphaneoides* spp. In the NE Pacific [37]. Small grenadiers (<20 cm TL) are rarely observed at baited cameras [38]. So while the video lander, traps and longlines may not sample the entire grenadier size distribution, all of these sampling approaches were baited so they are internally consistent.

### Nekton data

The California Cooperative Fisheries Investigation (CalCOFI) has conducted a comprehensive survey of fish larvae and eggs from the 1950′s to the present day from Baja, California north to the US-Canadian border (http://calcofi.org/). Hake and jack mackerel eggs hatch in about one day of being spawned [39] so the local abundance of eggs will positively correlate with local spawning biomass of adults. Egg abundance is used to assess spawning stock biomass in fisheries assessments [40], [41] including those for hakes [42], [43], [44]. There are no direct estimates of mortality during spawning for either hake or jack mackerel. In the absence of such data we must assume that mortality is constant over time for adult fish so that temporal variations in abundance of fish will be directly related to numbers of dying fish and sinking carcasses.

Several sampling types have been employed by the CalCOFI survey. Surface tows (Manta net) and vertical tows were excluded because hake and jack mackerel eggs were only identified and counted in these samples from 2006 to the present. An oblique bongo net system was used at all sampling stations and years and eggs were counted starting in 1988. This is a paired 71 cm diameter, 505 µm mesh net system. All tows were fished at 1–2 knots to 210 m depth. All data were standardized for the volume of water filtered. For squid, only *Loligo opalescens* paralarvae were counted. Gonatid squids brood their eggs in the meso and bathypelagic [45] thus their larvae are unlikely to appear in the CalCOFI samples and we could find no other alternative data sources with which to estimate their abundance off California.

**Figure 1:**
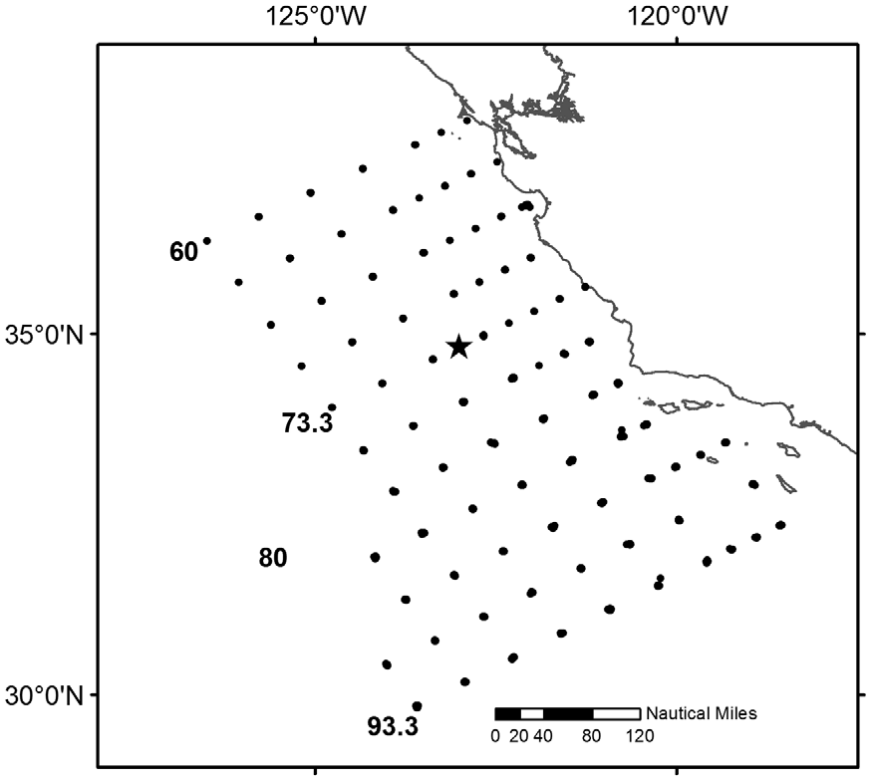
Stations used for analysis from the California Cooperative Fisheries Investigation (CalCOFI) fish egg surveys. The California coastline is depicted from San Diego in the south to San Francisco to the north. Several of the sampling line numbers are shown for reference. Station M is shown by the star.

The CalCOFI survey uses a regular suite of stations oriented in lines offshore in a southeasterly direction (330°) perpendicular to the California coastline. Lines are spaced 40 nmi apart in the offshore locations and stations are placed 20 to 40 nm apart along each line. Since 1985 the CalCOFI program regularly sampled from line 76.7 off central California to line 93.3 at the US-Mexican border (Figure 1). Lines 60–73.3 were sampled in some years and we analyzed the data for this larger area as well. Stations are occupied on each line from the nearshore environment to offshore several hundred km. We examined all stations on these lines offshore of the relatively shallow basins in between the Channel Islands (stations 45 and higher). We also examined the egg time series for all sampling stations within 100 nmi or 1.5 degrees of Station M (lines 70–80 and stations 60–90). Surveys were conducted quarterly and all data were converted to a 13 month moving average of egg abundance (# 1000 m^−3^) at each spatial scale.

The US National Marine Fisheries Service and Fisheries and Oceans Canada conduct annual stock assessments for pacific hake along the west coast of North America. This process synthesizes a considerable amount of data to generate models that estimate each year's total biomass and female spawning biomass among other variables [25]. Hake move offshore to spawn but the juveniles do not, so we used the female spawning stock biomass as the most appropriate index of potential carrion supply to grenadiers. The data we used were model median values.

Other time series of nekton abundance or community composition are available and have been summarized in Brodeur et al [46] based on coastal trawl and acoustic surveys. A pelagic trawl survey, focused on sampling young of the year rockfish, from Cape Mendocino south to San Diego covers the time period of interest [47]. However, it extends only 50 nmi offshore and data were for mobile 1 year old hake (no data for jack mackerel), thus the CalCOFI data seemed a better source of information. There is also a biennial acoustic survey along the US West coast to survey hake biomass [48]. Sampling is during the summer when the adults are feeding at the shelf break. The survey covers from Queen Charlotte Sound, Canada to 35.7° N (just north of Station M) and from the 50 m to the 1500 m isobath in transects every ∼20 km latitudinally (Fleischer et al 2005). So this time series does not characterize the hake population when it could be available to abyssal scavengers. Furthermore, during the survey season few hake are reported south of San Francisco. Due to the characteristics of these surveys they were not used to evaluate temporal patterns in nekton communities near Station M.

### Data analysis

Correlations were performed between grenadier and nekton egg monthly abundance using 13 month centered moving averages from the time series because they were discontinuous. Correlations with time lags of −12 to +12 months were used. Yearly estimates of hake female spawning stock biomass were also correlated to yearly averages of grenadier abundance. In this case no time lags were used because of the broad temporal scale of the measurements. To correct for serial autocorrelation the modified Chelton method was used [49]. Tests were considered significant if p<0.05. All statistical analysis was conducted using Statistica 7.1 software (Statsoft Inc., www.statsoft.com).

## Results

**Figure 2:**
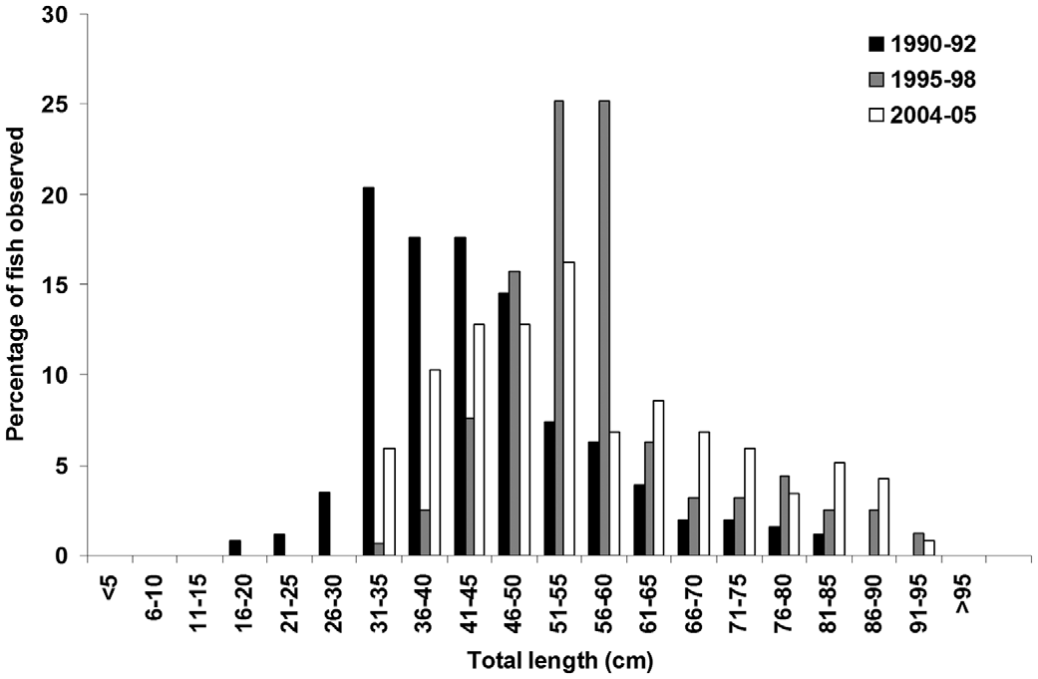
Size frequency distributions of *Coryphaenoides* spp. showing an increase in mean size from 1990–92 to 2004–2005.

In addition to previously documented increases in the abundance of grenadiers at Sta. M. [19], we show that their average size has increased compared to the earliest portion of the time series (ANOVA, F_2,473_ = 52, p<0.001, Figure 2). 197 fish measured in baited camera deployments from 1990–1992 had a mean total length (TL) of 46 cm. 159 fish measured from 1995–1998 had a mean TL of 59 cm similar to that measured from 2004–2005 (55 cm, 117 fish). This change in length from the first period to the last two is a change in mean mass of 0.4 to >0.8 kg based on length-weight relationships [37].

**Figure 3:**
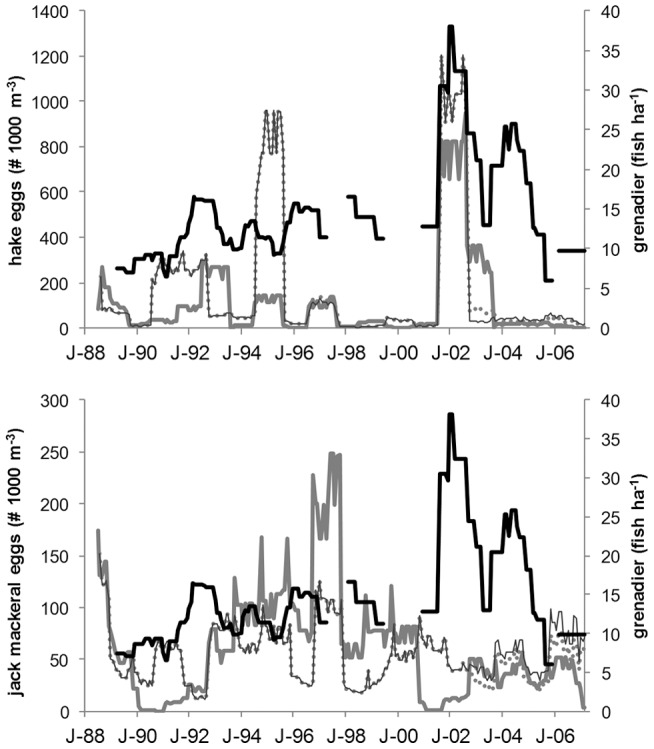
Time series of grenadier and epipelagic nekton egg abundances. Grenadier abundance (thick black line) and (a) hake and (b) jack mackerel egg abundance for the larger sampled area (gray dots), the annually sampled region (thin gray line) and only stations within 100 nmi of Station M (thick gray lines) are shown.

**Figure 4:**
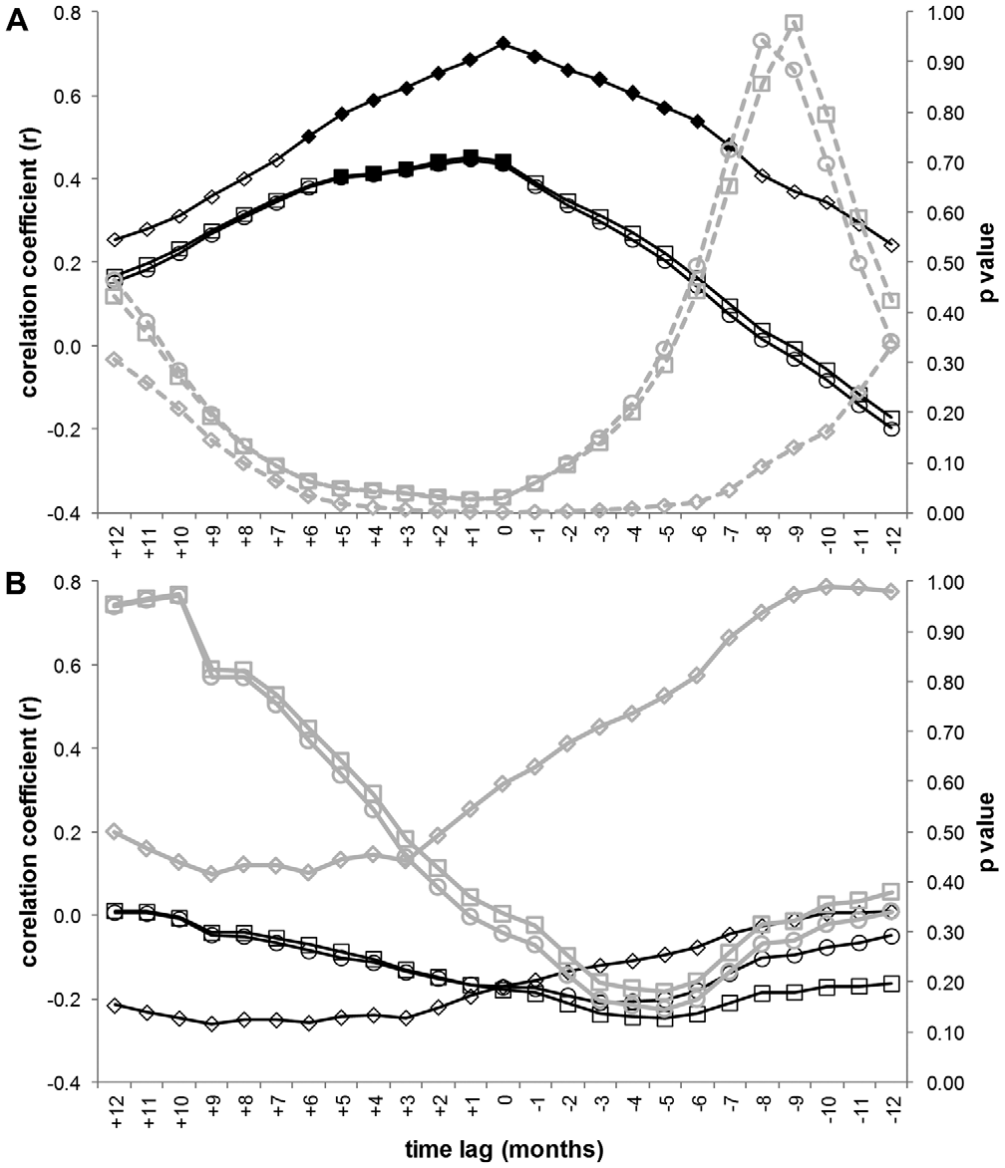
Correlation coefficients (black) and associate p values (grey) between grenadier and nekton egg abundances. Correlations were lagged from +12 (eggs leading grenadiers) and −12 (grenadiers leading eggs) months. Correlations to hake (A) and jack mackerel (B) from the 100 nmi (diamonds), regularly sampled area (circles) and larger area (squares) are shown. Solid black symbols for correlation coefficients indicate a significant correlation (p<0.05 as shown by the corresponding grey symbols).

Significant correlations between grenadier abundance and hake but not jack mackerel egg abundances were found (Figures 3 and 4). Grenadier abundance was negatively correlated with jack mackerel egg abundance over most of the time lags but not significantly so. In contrast, hake egg abundance was positively correlated with grenadier abundance at all spatial scales and stronger at the smaller 100 nmi spatial scale (Figure 4). At the larger spatial scales significant correlations (p<0.05) occurred when hake egg abundance led grenadier abundance by 0–5 months. At the smaller 100 nmi scale correlations were significant from +6 to −7 months. These correlations are influenced strongly by the coincidence in peak years (2001–2002) later in the time series though at the 100 nmi scale the fluctuations in both grenadier and hake egg density are also significantly correlated during the early to mid 1990′s (Figure 3).

**Figure 5:**
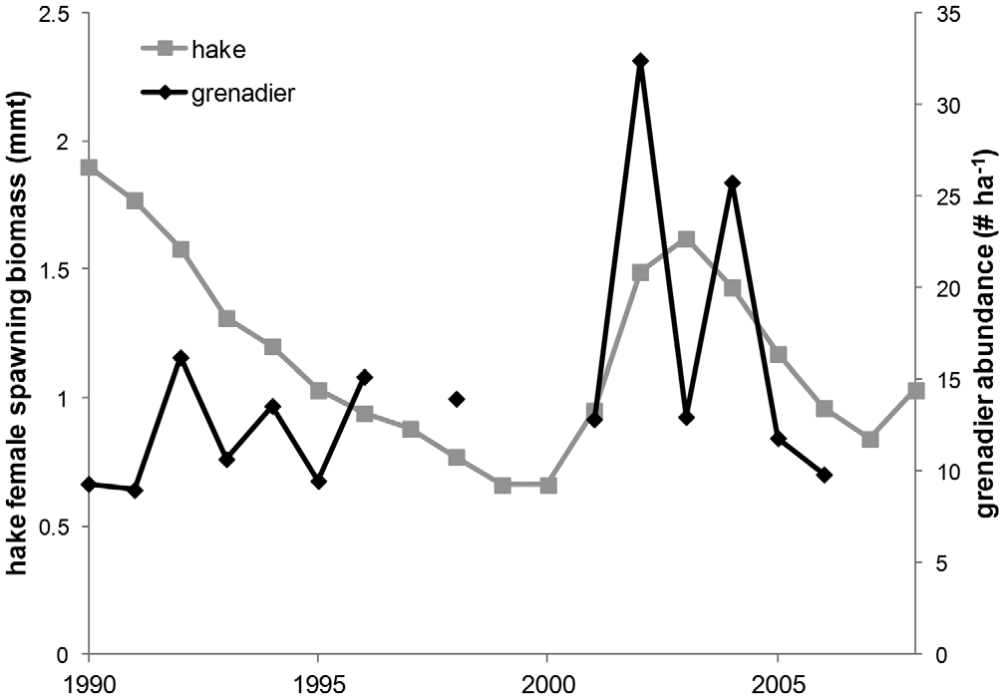
Time series of yearly spawning stock biomass of hake [**25**] and yearly mean grenadier abundance at Sta. M.

Correlations between yearly estimates of hake female spawning stock biomass along the west coast of the North America and grenadier abundance at Station M towards the southern extent of the hake stock were not significant. Although when grenadier abundance rose at Sta. M in the early 2000′s, hake biomass rose concurrently (Figure 5).

## Discussion

The changes in grenadier population structure followed metrics for hake biomass, suggesting that these abyssal fishes respond rapidly to changes in epipelagic carrion sources. Rapid consumption once carrion reaches the seafloor [29], [30], [31] may explain the small temporal lags (Figure 4) between the abyssal and epipelagic fish populations at Sta. M. In contrast to the results for hake, correlations to jack mackerel egg density were negative and insignificant (Fig. 3). This species has much less biomass in the California Current ecosystem compared to hake [46] and is widely distributed without a pronounced spawning period [50], [51], [52] which may explain the difference.

Our results show that there was both an increase in abundance of grenadiers and an increase in mean size such that fish biomass at Station M increased ∼6 fold. Almost certainly the changes are the result of migration rather than local individual and population growth because of the slow growth rates of grenadiers [von Bertalanfy k = 0.02–0.11 and longevity 25–73 years; 20,21,53]. Migration rates of 250 km month^−1^ are possible given grenadiers slow (∼0.1 m s^−1^) continuous swimming [54], [55]. Studies of abyssal grenadiers in the North Atlantic and in the central North Pacific gyre also observed seasonal or interannual changes in lengths attributing them to migration rather than local population effects [56], [57].

While migration is the most likely explanation for the current results, grenadier reproduction and/or recruitment variation is an alternative explanation. Increases in the mean size of grenadiers and the absence of the smallest individuals (Figure 2) in the latter sampling periods could be explained by a recruitment pulse which then moved through the population, perhaps affecting abundance patterns as well. It has been suggested that deep-sea fishes [58] and other animals [17] have sporadic recruitment events. However, this is unlikely in the case of the grenadiers at Sta. M for several reasons. The changes in size that were observed are not monotonic with time. The mean size peaked in the 1995–1998 sample (Figure 2). Also, the change in size likely represents a longer duration of growth than the time elapsed. There are no validated age and growth estimates for *C. armatus*, however, we do know that other related species in the genus grow very slowly. A recruitment pulse is also unlikely because the expectation would be an increase in mean size over time as a good year class moves through the population but the abundance would also be expected to decline through natural mortality. A recruitment pulse is also unlikely to explain our results given the apparently localised area where the correlation to hake egg abundance was significant.

The location of hake spawning probably is more important than the size of the spawning stock in understanding the dynamics of abyssal grenadier populations. The correlation to hake egg density is stronger for data from a more localized region (100 nmi), probably because carrion rapidly settles to the seafloor. Close spatial coupling is also supported by the finding that hake spawning stock biomass for the whole California Current system showed no significant relationship to grenadier abundance. Hake spawn principally off central and southern California but can extend off shore of Baja California, Mexico and as far north as Oregon [26], [27], [59]. The CalCOFI data set's regular sampling grid only covers the middle of this range limiting our ability to quantify variations in spawning location. However, coastal trawl and acoustic surveys provide a qualitative comparison to the grenadier data. They inferred more spawning activity north of California in the late 1990 s from the presence of pre-recruits and observed a southern shift in the early 2000′s due to a cold water La Nina event [25], [27]. This broad pattern is coincident with the increase in grenadier abundance at Station M. In spring and summer, hake move inshore and north to feed and a recent study has shown that the extent of these movements is affected interannually by variation in along-slope California undercurrent flow [26]. Thus it is interesting to speculate that the grenadiers might show a similar migration pattern on annual or interannual timescales. Not all peaks in hake egg density correspond to a peak in grenadier abundance at Sta. M suggesting other factors are also important. It may be that the pattern we see at Sta. M is part of a larger heterogenous and changing distribution of grenadiers on the abyssal floor which is imperfectly captured at our single study location.

A growing body of literature is illustrating the ways in which climate, surface productivity and export flux control abyssal ecosystem function [16], [17], [60], [61]. For the grenadiers, these indices do not correlate to their population dynamics but proxies for carrion flux and epibenthic megafaunal abundance both do [19]. The correlations between grenadier and megafaunal abundance were lagged by 9–20 months, whereas correlations between grenadier and hake showed lags of less than ∼6 months, suggesting that the grenadiers respond first to trophically more important carrion [1], [22]. This implies that a full understanding of abyssal ecosystem dynamics needs to incorporate knowledge of epipelagic nekton as well as primary producers.

Climate and fishing pressures could modulate deep-sea fish dynamics. Climate change induced surface warming may reduce epipelagic nekton biomass but due to their mobility can also cause shifts in their distributions and changes in community composition [23], [46], [62], [63]. Studies have suggested that fish will move north [62] and closer to the coast [46], [63] as low productivity regions expand in the gyres. *C. yaquinae* is limited to the North Pacific central gyre at depths deeper than 4000 m and may not be able to follow [64]. *C. armatus* is found shallower, up to ∼3000 m and worldwide [57], so might be less limited in its movement ability. Fishing activities in surface waters likely have little influence on vertical small particle flux and thus on most of the abyssal benthic community but our results suggest they could affect deep-sea scavengers, greatly extending the area and vertical extent of fishing influence. In the short term, fishing can increase carrion flux as a result of discarded bycatch [65], [66]. Fisheries offal has been noted in the stomachs of deep-sea fishes [67], [68]. In the long term, global reductions in fish stocks [69] should reduce carrion flux [70]. Decreases in whale carrion flux from commercial whaling in the 20^th^ century may have caused extinctions of endemic whale fall invertebrates [71]. However, in the Atlantic, while Bailey et al [72] showed changes in fish abundance below the depths reached by fishing boats, known scavengers were no more likely to change in abundance than non-scavengers.

Other species of deep-sea fish could be influenced by the dynamics of epipelagic fish stocks because many feed on carrion [67], [73], [74], [75], [76], [77], [78], [79], [80] or are strongly attracted to bait [29], [30], [38], [55], [81], [82], [83], [84], [85], [86], [87], [88]. Many of these fishes live in areas where there are seasonal variations in abundance of epipelagic species due to migration (i.e. albacore [89] or whales [32]) or spawning aggregations (i.e. blue whiting or hoki [90], [91]). Future studies of the deep-sea ecosystem should incorporate an understanding of the status and trends of nekton populations. Although time series of abyssal fish populations are rare [the only other is in the North Atlantic; 72], there is more data for the continental slopes. These systems are directly affected by fisheries complicating interpretation of population changes but opportunities exist to evaluate how pelagic fish dynamics effect deep-sea demersal fish populations.

## References

[1] Drazen JC, Popp BN, Choy CA, Clemente T, De Forest LG, et al (2008) Bypassing the abyssal benthic food-web: macrourid diet in the eastern North Pacific inferred from stomach content and stable isotopes analyses. Limnology and Oceanography 53: 2644–2654.

[2] Pearcy WG, Ambler JW (1974) Food habits of deep-sea fishes off the Oregon coast. Deep Sea Research 21: 745–759.

[3] Gartner JV, Crabtree RE, Sulak KJ (1997) Feeding at depth. In: Randall DJ, Farrell AP, editors. Deep-sea fishes. San Diego: Academic Press. 115–193.

[4] Crabtree RE, Carter J, Musick JA (1991) The comparative feeding ecology of temperate and tropical deep-sea fishes from the western North Atlantic. Deep Sea Research 38: 1277–1298.

[5] Worm B, Barbier EB, Beaumont N, Duffy E, Folke C, et al (2006) Impacts of Biodiversity Loss on Ocean Ecosystem Services. Science 314: 787–790. 17082450 10.1126/science.1132294

[6] Myers RA, Baum JK, Shepherd TD, Powers SP, Peterson CH (2007) Cascading Effects of the Loss of Apex Predatory Sharks from a Coastal Ocean. Science 315: 1846–1850. 17395829 10.1126/science.1138657

[7] Heithaus MR, Frid A, Wirsing AJ, Worm B (2008) Predicting ecological consequences of marine top predator declines. Trends in Ecology & Evolution 23: 202–210. 18308421 10.1016/j.tree.2008.01.003

[8] Baum JK, Worm B (2009) Cascading top-down effects of changing oceanic predator abundances. Journal of Animal Ecology: 1–16. 10.1111/j.1365-2656.2009.01531.x 19298616

[9] Estes JA, Terborgh J, Brashares JS, Power ME, Berger J, et al (2011) Trophic downgrading of planet Earth. Science 333: 301–306. 21764740 10.1126/science.1205106

[10] Chavez FP, Ryan J, Lluch-Cota SE, Niquen C M (2003) From anchovies to sardines and back: multidecadal change in the Pacific Ocean. Science 299: 217–221. 12522241 10.1126/science.1075880

[11] Hare SR, Mantua NJ (2000) Empirical evidence for North Pacific regime shifts in 1977 and 1989. Progress in Oceanography 47: 103–145.

[12] Beaugrand G, Brander KM, Lindley JA, Souissi S, Reid PC (2003) Plankton effect on cod recruitment in the North Sea. Nature 426: 661–664. 14668864 10.1038/nature02164

[13] Smith KL Jr, Druffel ERM (1998) Long time-series monitoring of an abyssal site in the NE Pacific: an introduction. Deep Sea Research II 45: 573–586.

[14] Smith KL Jr, Ruhl HA, Kaufmann RS, Kahru M (2008) Tracing abyssal food supply back to upper-ocean processes over a 17-year time series in the northeast Pacific Limnology and Oceanography. 53: 2655–2667.

[15] Drazen JC, Baldwin RJ, Smith KL Jr (1998) Sediment community response to a temporally varying food supply at an abyssal station in the NE Pacific. Deep Sea Research II 45: 893–913.

[16] Ruhl HA, Ellena JA, Smith KL Jr (2008) Connections between climate, food limitation, and carbon cycling in abyssal sediment communities. Proceedings of the National Academy of Sciences 105: 17006–17011. 10.1073/pnas.0803898105 PMC2579368 18974223

[17] Ruhl HA (2007) Abundance and size distribution dynamics of abyssal epibenthic megafauna in the northeast Pacific. Ecology 88: 1250–1262. 17536411 10.1890/06-0890

[18] Ruhl HA, Smith KL Jr (2004) Shifts in deep-sea community structure linked to climate and food supply. Science 305: 513–515. 15273392 10.1126/science.1099759

[19] Bailey DM, Ruhl HA, Smith KL Jr (2006) Long-term change in benthopelagic fish abundance in the abyssal N.E. Pacific Ocean Ecology. 87: 549–555. 10.1890/04-1832 16602284

[20] Lorance P, Garren F, Vigneau J (2003) Age estimation of roundnose grenadier (Coryphaenoides rupestris), effects of uncertainties on ages. Journal of Northwest Atlantic Fisheries Science 31: 387–399.

[21] Andrews AH, Cailliet GM, Coale KH (1999) Age and growth of the Pacific grenadier (*Coryphaenoides acrolepis*) with age estimate validation using an improved radiometric ageing technique. Canadian Journal of Fisheries and Aquatic Sciences 56: 1339–1350.

[22] Drazen JC, Phleger CF, Guest MA, Nichols PD (2009) Lipid compositions and diet inferences of abyssal macrourids in the eastern North Pacific Marine Ecology Progress Series. 387: 1–14.

[23] Zeidberg LD, Robison BH (2007) Invasive range expansion by the Humboldt squid, *Dosidicus gigas*, in the eastern North Pacific. Proceedings of the National Academy of Sciences of the United States of America 104: 12948–12950. 17646649 10.1073/pnas.0702043104 PMC1937572

[24] Brodeur RD, Fisher JP, Emmett RL, Morgan CA, Casillas E (2005) Species composition and community structure of pelagic nekton off Oregon and Washington under variable oceanographic conditions. Marine Ecology Progress Series 298: 41–57.

[25] Stewart IJ, Forrest RE, Grandin C, Hamel OS, Hicks AC, et al. (2011) Status of the Pacific Hake (Whiting) stock in U.S. and Canadian Waters in 2011. Joint US and Canadian Hake Technical Working Group Report: 1–207.

[26] Agostini VN, Francis RC, Hollowed AB, Pierce SD, Wilson C, et al (2006) The relationship between Pacific hake (Merluccius productus) distribution and poleward subsurface flow in the California Current System. Canadian Journal of Fisheries and Aquatic Sciences 63: 2648–2659.

[27] Ressler PH, Holmes JA, Fleischer GW, Thomas RE, Cooke KC (2008) Pacific Hake, *Merluccius productus*, Autecology: A Timely Review. Marine Fisheries Review 69: 1–24.

[28] Priede IG, Bagley PM, Armstrong JD, Smith KL Jr, Merrett NR (1991) Direct measurement of active dispersal of food-falls by deep-sea demersal fishes. Nature 351: 647–649.

[29] Yeh J, Drazen JC (2011) Baited camera observations of megafaunal scavenger ecology of the California slope. Marine Ecology Progress Series 424: 145–156.

[30] Soltwedel T, von Juterzenka K, Premke K, Klages M (2003) What a lucky shot! Photographic evidence for a medium-sized natural food-fall at the deep seafloor. Oceanologica Acta 26: 623–628.

[31] Stockton WL, DeLaca TE (1982) Food falls in the deep sea: occurrence, quality, and significance. Deep Sea Research 29: 157–169.

[32] Smith CR, Baco AR (2003) Ecology of whale falls at the deep-sea floor. Oceanography and Marine Biology: An Annual Review 41: 311–354.

[33] Lauerman LML, Kaufmann RS, Smith KL Jr (1996) Distribution and abundance of epibenthic megafauna at a long time-series station in the abyssal Northeast Pacific. Deep Sea Research I 43: 1075–1103.

[34] Priede IG, Bagley PM, Smith KL Jr (1994) Seasonal change in activity of abyssal demersal scavenging grenadiers *Coryphaenoides (Nematonurus) armatus* in the eastern North Pacific Ocean. Limnology and Oceanography 39: 279–285.

[35] Bailey DM, Bagley PM, Jamieson AJ, Collins MA, Priede IG (2003) In situ investigation of burst swimming and muscle performance in the deep-sea fish *Antimora rostrata* . Journal of Experimental Marine Biology and Ecology 285–286: 295–311.

[36] Drazen JC (2002) A seasonal analysis of the nutritional condition of deep-sea macrourid fishes in the north-east Pacific. Journal of Fish Biology 60: 1280–1295.

[37] Drazen JC (2002) Energy budgets and feeding rates of *Coryphaenoides acrolepis* and *C. armatus* . Marine Biology 140: 677–686.

[38] King NJ, Bagley PM, Priede IG (2006) Depth zonation and latitudinal distribution of deep-sea scavenging demersal fishes of the Mid-Atlantic Ridge, 42 to 53°N. Marine Ecology Progress Series 319: 263–274.

[39] Pauly D, Pullin RSV (1988) Hatching time in spherical, pelagic, marine fish eggs in response to temperature and egg size. Environmental biology of fishes 22: 261–271.

[40] Koslow JA, Bulman CM, Lyle JM, Haskard KA (1995) Biomass assessment of a deep-water fish, the orange roughy (*Hoplostethus atlanticus*), based on an egg survey. Marine & Freshwater Research 46: 819–830.

[41] Zeldis JR (1993) Applicability of egg surveys for spawning-stock biomass estimation of snapper, orange roughy, and hoki in New Zealand. Bulletin of Marine Science 53: 864–890.

[42] Mason JC (1986) Fecundity of the Pacific hake, *Merluccius productus*, spawning in Canadian waters. Fishery Bulletin 84: 209–216.

[43] Mehault S, Dominguez-Petit R, Cervino S, Saborido-Rey F (2010) Variability in total egg production and implications for management of the southern stock of European hake. Fisheries Research 104: 111–122.

[44] Murua H, Ibaibarriaga L, Alvarez P, Santos M, Korta M, et al (2010) The daily egg production method: A valid tool for application to European hake in the Bay of Biscay? Fisheries Research 104: 100–110.

[45] Seibel BA, Robison BH, Haddock SHD (2005) Post-spawning egg care by a squid. Nature 438: 929. 16355206 10.1038/438929a

[46] Brodeur RD, Pearcy WG, Ralston S (2003) Abundance and distribution patterns of nekton and micronekton in the northern California current transition zone. Journal of Oceanography 59: 515–535.

[47] Sakuma KM, Ralston S, Wespestad VG (2006) Interannual and spatial variation in the distribution of young-of-the-year rockfish (*Sebastes* spp): expanding and coordinating a survey sampling frame. CalCOFI Report 47: 127–139.

[48] Fleischer GW, Cooke KD, Ressler PH, Thomas RE, Blois SKd, et al (2005) The 2003 integrated acoustic and trawl survey of pacific hake, *Merluccius productus*, in U.S. and Canadian waters off the Pacific coast. US Dept Commer, NOAA Tech Memo NMFS-NWFSC 65: 1–45.

[49] Pyper BJ, Peterman RM (1998) Comparison of methods to account for autocorrelation in correlation analyses of fish data. Canadian Journal of Fisheries and Aquatic Sciences 55: 2127–2140.

[50] MacCall AD, Stauffer GD (1983) Biology and fishery potential of jack mackerel (*Trachurus symmetricus*). CalCOFI Report 24: 46–56.

[51] Macewicz BJ, Hunter JR (1993) Spawning frequency and batch fecundity of jack mackerel, *Trachurus symmetricus*, off California during 1991. CalCOFI Report 34: 112–121.

[52] Theilacker GH (1985) Starvation-induced mortality of young sea-caught jack mackerel, Trachurus symmetricus, determined with histological and morphological methods. Fishery Bulletin 84: 1–17.

[53] Drazen JC, Haedrich RL (2012) A continuum of life histories in deep-sea demersal fishes. Deep Sea Research I 61: 34–42.

[54] Ruxton GD, Bailey DM (2005) Searching speeds and the energetic feasibility of an obligate whale-scavenging fish. Deep Sea Research I 52: 1536.

[55] Priede IG, Bagley PM (2000) In situ studies on deep-sea demersal fishes using autonomous unmanned ladder platforms. Oceanography and Marine Biology: An Annual Review 38: 357–392.

[56] Priede IG, Deary AR, Bailey DM, Smith KL Jr (2003) Low activity and seasonal change in population size structure of grenadiers in the oligotrophic abyssal Central North Pacific Ocean. Journal of Fish Biology 63: 187–196.

[57] King NJ, Priede IG (2008) Coryphaenoides armatus, the Abyssal Grenadier: Global Distribution, Abundance, and Ecology as Determined by Baited Landers. In: Orlov AM, Iwamoto T, editors. Grenadiers of the World Oceans: Biology, Stock Assessment, and Fisheries. Bethesda, MD: American Fisheries Society. 139–161.

[58] Francis CRIC, Clark MR (2005) Sustainability issues for orange roughy fisheries. Bulletin of Marine Science 76: 337–352.

[59] Horne JK, Smith PE (1997) Space and time scales in pacific hake recruitment processes: Latitudinal variation over annual cycles. Reports of California Cooperative Oceanic Fisheries Investigations 38: 90–102.

[60] Smith KL Jr, Ruhl HA, Bett BJ, Billett DSM, Lampitt RS, et al (2009) Climate, carbon cycling, and deep-ocean ecosystems. Proceedings of the National Academy of Sciences of the United States of America 106: 19211–19218. 19901326 10.1073/pnas.0908322106 PMC2780780

[61] Smith CR, De Leo FC, Bernardino AF, Sweetman AK, Arbizu PM (2008) Abyssal food limitation, ecosystem structure and climate change. Trends in Ecology & Evolution 23: 518. 18584909 10.1016/j.tree.2008.05.002

[62] Perry AL, Low PJ, Ellis JR, Reynolds JD (2005) Climate change and distribution shifts in marine fishes. Science 308: 1912–1915. 15890845 10.1126/science.1111322

[63] Polovina JJ, Dunne JP, Woodworth PA, Howell EA (2011) Projected expansion of the subtropical biome and contraction of the temperate and equatorial upwelling biomes in the North Pacific under global warming. ICES Journal of Marine Science 68: 986–995.

[64] Wilson RR Jr, Waples RS (1983) Distribution, morphology, and biochemical genetics of *Coryphaenoides armatus* and *C. yaquinae* (Pisces: Macrouridae) in the central and eastern North Pacific. Deep Sea Research 30: 1127–1145.

[65] Kaiser MJ, Hiddink JG (2007) Food subsidies from fisheries to continental shelf benthic scavengers. Marine Ecology Progress Series 350: 267–276.

[66] Catchpole TL, Frid CLJ, Gray TS (2006) Importance of discards from the English *Nephrops norvegicus* fishery in the North Sea to marine scavengers. Marine Ecology Progress Series 313: 215–226.

[67] Drazen JC, Buckley TW, Hoff GR (2001) The feeding habits of slope dwelling macrourid fishes in the eastern North Pacific. Deep Sea Research I 48: 909–935.

[68] Laptikhovsky V, Fetisov A (1999) Scavenging by fish of discards from the Patagonian squid fishery. Fisheries Research 41: 93–97.

[69] Pauly D, Christensen V, Guenette S, Pitcher TJ, Sumaila UR, et al (2002) Towards sustainability in world fisheries. Nature 418: 689–695. 12167876 10.1038/nature01017

[70] Wilson EE, Wolkovich EM (2011) Scavenging: how carnivores and carrion structure communities. Trends in Ecology & Evolution 26: 129–135. 21295371 10.1016/j.tree.2010.12.011

[71] Smith CR (2006) Bigger Is Better: The Role of Whales as Detritus in Marine Ecosystems. Whales, Whaling and Ocean Ecosystems: UC Press. 284–298.

[72] Bailey DM, Collins MA, Gordon JDM, Zuur AF, Priede IG (2009) Long-term changes in deep-water fish populations in the northeast Atlantic: a deeper reaching effect of fisheries? Proceedings of the Royal Society B: Biological Sciences 276: 1965–1969. 19324746 10.1098/rspb.2009.0098 PMC2677247

[73] Britton JC, Morton B (1994) Marine carrion and scavengers. Oceanography and Marine Biology: An Annual Review 32: 369–434.

[74] Martin B, Christiansen B (1997) Diets and standing stocks of benthopelagic fishes at two bathymetrically different midoceanic localities in the Northeast Atlantic. Deep Sea Research I 44: 541–558.

[75] Bjelland O, Bergstad OA, Skjaeraasen JE, Meland K (2000) Trophic ecology of deep-water fishes associated with the continental slope of the eastern Norwegian Sea. Sarsia 85: 101–117.

[76] Anderson M (2005) Food habits of some deep-sea fish off South Africa's west coast and Agulhas Bank. 2. Eels and spiny eels African Journal of Marine Science 27: 557–566.

[77] Jones MRL (2008) Dietary analysis of *Coryphaenoides serrulatus, C. subserrulatus* and several other species of macrourid fish (Pisces: Macrouridae) from northeastern Chatham Rise, New Zealand. New Zealand Journal of Marine and Freshwater Research 42: 73–84.

[78] Boyle MD, Ebert DA, Cailliet GM (2012) Stable-isotope analysis of a deep-sea benthic-fish assemblage: evidence of an enriched benthic food web. Journal of Fish Biology 80: 1485–1507. 22497394 10.1111/j.1095-8649.2012.03243.x

[79] Clarke MR, Merrett N (1972) The significance of squid, whale and other remains from the stomachs of bottom-living deep-sea fish. Journal of the Marine Biological Association of the United Kingdom 52: 599–603.

[80] Mauchline J, Gordon JDM (1984) Feeding and bathymetric distribution of the gadoid and morid fish of the Rockall Trough. Journal of the Marine Biological Association of the United Kingdom 64: 657–665.

[81] Collins MA, Yau C, Nolan CP, Bagley PM, Priede IG (1999) Behavioural observations on the scavenging fauna of the Patagonian slope. Journal of the Marine Biological Association of the United Kingdom 79: 963–970.

[82] Witte U (1999) Consumption of large carcasses by scavenger assemblages in the deep Arabian Sea: Observations by baited camera. Marine Ecology Progress Series 183: 139–147.

[83] Yeh J, Drazen JC (2009) Depth zonation and bathymetric trends of deep-sea megafaunal scavengers of the Hawaiian Islands. Deep Sea Research I 56: 251–266.

[84] Jamieson AJ, Kilgallen NM, Rowden AA, Fujii T, Horton T, et al (2011) Bait-attending fauna of the Kermadec Trench, SW Pacific Ocean: Evidence for an ecotone across the abyssal-hadal transition zone. Deep Sea Research Part I: Oceanographic Research Papers 58: 49–62.

[85] Priede IG, Godbold JA, King NJ, Collins MA, Bailey DM, et al (2010) Deep-sea demersal fish species richness in the Porcupine Seabight, NE Atlantic Ocean: global and regional patterns. Marine Ecology 31: 247–260.

[86] Isaacs JD, Schwartzlose RA (1975) Active animals of the deep-sea floor. Scientific American 233: 85–91.

[87] Jones EG, Tselepides A, Bagley PM, Collins MA, Priede IG (2003) Bathymetric distribution of some benthic and benthopelagic species attracted to baited cameras and traps in the deep eastern Mediterranean. Marine Ecology Progress Series 251: 75–86.

[88] King NJ, Jamieson AJ, Bagley PM, Priede IG (2008) Deep-sea scavenging demersal fish fauna of the Nazare Canyon system, Iberian coast, north-east Atlantic Ocean. Journal of Fish Biology 72: 1804–1814.

[89] Beamish RJ, McFarlane GA, King JR (2005) Migratory patterns of pelagic fishes and possible linkages between open ocean and coastal ecosystems off the Pacific coast of North America. Deep Sea Research II 52: 739.

[90] Gunn JS, Bruce BD, Furlani DM, Thresher RE, Blaber SJM (1989) Timing and location of spawning of blue grenadier, Macruronus novaezelandiae (Teloestei: Merlucciidae), in Australian coastal waters. Australian Journal of Marine and Freshwater Research 40: 97–112.

[91] Ryan AW, Mattiangeli V, Mork J (2005) Genetic differentiation of blue whiting (*Micromesistius poutassou* Risso) populations at the extremes of the species range and at the Hebrides–Porcupine Bank spawning grounds. ICES Journal of Marine Science 62: 948–955.

